# The impact of intermittent fasting during weight reduction in people living with type 2 diabetes mellitus: a randomized clinical trial

**DOI:** 10.1038/s41430-025-01693-z

**Published:** 2026-01-07

**Authors:** Salma M. Abdel Fattah, Maggie M. Abbassi, Samah Abd Elshafy, Mona A. Hegazy, Samar F. Farid

**Affiliations:** 1 Department of Clinical Pharmacy, Faculty of Pharmacy, Cairo University, Cairo, Egypt; 2 Diabetes and Endocrinology Nutrition Clinic, Cairo University Hospitals (Kasr Alainy), Cairo, Egypt; 3 Department of Internal Medicine, Faculty of Medicine, Cairo University, Cairo, Egypt

**Keywords:** Type 2 diabetes, Obesity

## Abstract

**Objectives:**

Intermittent fasting (IF) has gained attention for weight management and metabolic health, but detailed trials comparing its efficacy to calorie restriction (CR) in type 2 diabetes mellitus (T2D) are limited. Our aim was to compare the impact of CR diet with or without 12-hour overnight IF regimen on weight loss, glycemic control, and reduction of medication use in T2D.

**Methods:**

This was a 3-month, single-center randomized clinical trial. Participants (N = 99), 21-60 years with T2D, were randomly assigned to Group 1 (12-hour IF + CR, N = 48) or Group 2 (CR alone, N = 51). Anthropometric evaluations included weight, height, waist and hip circumference, and BMI. Body composition analysis was performed, and biochemical analysis included HbA1c, FBG, and fasting insulin levels to calculate the HOMA-IR. Physical activity was assessed using the Global Physical Activity Questionnaire.

**Results:**

Both groups experienced significant weight loss after 12 weeks. The IF + CR group showed a more pronounced weight reduction (-6.51%) compared to the CR group (-4.41%) (P < 0.001) and significant improvements in HbA1c levels, with a reduction to 6.51% compared to 6.86% in the CR group (P = 0.035). The absolute reduction in HbA1c showed a significantly greater median decrease in the IF + CR group [−0.50 (IQR − 0.60 to −0.35)] compared with the CR group [−0.20 (IQR − 0.40 to −0.10)] (P = 0.002). IF + CR demonstrated significant reductions in waist circumference (-4.64%) and hip circumference (-3.12%) compared to the CR (-2.70% and -0.86%, respectively) (P < 0.05). Furthermore, IF + CR showed greater reductions in body fat mass (-9.39%) compared to the CR (-5.32%) (P < 0.001). Physical activity levels were comparable, with average MET of 100 and sedentary hours of 15.7 ( ± 3) for IF + CR and 16.2 ( ± 2.3) for CR, (P = 0.134).

**Conclusions:**

A 12-hour intermittent fasting (IF) regimen combined with calorie restriction (CR) demonstrated superior efficacy in promoting weight loss and improving glycemic control in patients with type 2 diabetes (T2D) compared to CR alone. Integrating IF into clinical nutrition guidelines could offer a practical and effective approach to diabetes management.

**Clinical trial registration:**

The study was registered at the Pan African Clinical Trial Registry (PACTR202411524439559).

## Introduction

Diabetes mellitus, a chronic metabolic disorder characterized by persistent hyperglycemia due to impairments in insulin secretion, insulin sensitivity, or both, has escalated to pandemic proportions [1]. In 2024, the global prevalence of diabetes among adults exceeded 800 million, posing a significant challenge to healthcare systems worldwide [2]. This condition is a predominant cause of morbidity and mortality, significantly worsening the risk of cardiovascular disease, renal failure, and lower-limb amputations [3]. Furthermore, diabetes imposes a substantial economic burden, with worldwide healthcare expenditures anticipated to reach $1 trillion by 2045 [4].

Diabetes is a significant public health issue in Egypt, with a substantial impact on healthcare expenditure. As of recent estimates, the total diabetes-related health expenditure in Egypt is estimated to reach approximately $4.48 billion by 2045 [5].

Effective management of diabetes focuses on dietary interventions aimed at regulating blood glucose levels and preventing associated complications [1]. Traditional dietary strategies, such as low-carbohydrate diets, portion control, and balanced nutrition, focus on optimizing glycemic control [1]. However, recent interest has shifted toward alternative dietary approaches like intermittent fasting (IF) and calorie restriction (CR), which have demonstrated promising metabolic benefits [6].

Intermittent fasting, which alternates between periods of eating and fasting, and calorie restriction, defined by sustained reductions in caloric intake without malnutrition, have garnered increasing attention for their potential to improve insulin sensitivity, glycemic control, and weight management in diabetes patients [7, 8]. Despite the potential benefits, evidence remains inconclusive, with ongoing debates about their effectiveness, sustainability, and safety in diabetic populations [9].

Given this uncertainty, a comparative evaluation of IF and CR is critical [10]. While CR is well-established for its consistent benefits in weight reduction and enhancing life span, IF offers periodic dietary restrictions, which may be more sustainable for certain individuals [11]. Both approaches hold the potential to enhance glycemic control, improve insulin sensitivity, and positively influence body composition [12]. Our aim was to compare the impact of CR diet with or without 12-hour IF regimen on weight loss, glycemic control and reduction of medication use over a three-month period, to provide evidence-based recommendations for optimizing dietary management in diabetes care.

## Methods

### Study design

This was a randomized, open label, controlled clinical trial conducted at Faculty of Medicine Cairo University hospitals outpatient diabetes and endocrinology nutrition clinic from November 2021 to May 2024. The protocol of our study received approval from the Research and Ethics Committee for Experimental and Clinical Studies, Faculty of Pharmacy, Cairo University CL (2987) (Approval date: April 2021). Written informed consent was obtained from all participants prior to enrollment. The trial was registered at the Pan African Clinical Trial Registry (PACTR202411524439559) and conducted following the Declaration of Helsinki [13].

### Participants

Eligible participants were adults aged 21 to 60 years, diagnosed with type 2 DM, and classified as obese with a body mass index (BMI) ranging from 30 to 50 kg/m². All participants had been on metformin only therapy for at least one year. Exclusion criteria included individuals undergoing insulin therapy, those receiving other antidiabetic medications (e.g., sulfonylureas, DPP-4 inhibitors, GLP-1 receptor agonists, SGLT2 inhibitors, or thiazolidinediones), or those diagnosed with type 1 diabetes. Additionally, those engaged in high-intensity endurance exercises (defined as sustained, vigorous activities exceeding 60 minutes) were excluded. Participants with significant comorbidities that could impede adherence to dietary interventions were also excluded. These comorbidities included severe cardiovascular conditions (e.g., heart failure, myocardial infarction), respiratory diseases (e.g., chronic obstructive pulmonary disease), psychiatric disorders (e.g., major depressive disorder, schizophrenia), musculoskeletal disorders (e.g., rheumatoid arthritis), individuals with chronic liver disease, severe renal impairment (GFR < 30 mL/min/1.73 m²), or thyroid disorders. Those receiving treatment with thyroxine, antithyroid medications, or immunosuppressive agents were deemed ineligible. Additional exclusion criteria included pregnancy, and alcoholism. Also, individuals unable to provide informed consent due to cognitive impairment were excluded.

### Intervention and outcomes

Eligible participants (N = 125) were randomly assigned to one of two study groups using computer-generated random numbers (Research Randomizer version 4.0). Block randomization with a block size of 4 was used in a 1:1 ratio to maintain balanced group sizes [14]. Group 1 (N = 48) followed a 12-hour night intermittent fasting regimen combined with calorie restriction, while Group 2 (N = 51) followed a calorie-restricted diet only (CR) for 12 weeks. Participants in Group 1 (night IF combined with CR) adhered to a 12-hour fasting regimen, abstaining from food from 8:00 PM to 8:00 AM daily. This modification was implemented to suit cultural and practical considerations in Egypt, promoting better adherence to the intervention. During the fasting period, participants were encouraged to consume plenty of water. In Group 2 (Calorie Restriction), participants were provided with a caloric deficit diet plan designed by a nutritionist, without fasting windows. Participants in both groups followed the same macronutrient distribution: 45% carbohydrates, 20% protein, and 35% fats [15]. Caloric intake for both groups was personalized using the Mifflin-St Jeor equation, which calculates individual energy needs based on age, gender, weight, and height, and adjusts for sedentary activity levels. A standardized calorie deficit of 500 kcal was then applied to each participant’s calories to ensure consistency in energy restriction across the groups [16, 17].

The primary outcome of the study was the absolute change in glycated hemoglobin (HbA1c) levels, given its status as a clinically meaningful indicator of glycemic control in individuals with type 2 diabetes. Secondary outcomes were categorized into efficacy and safety outcomes. Efficacy outcomes included changes in body weight, insulin resistance as measured by HOMA-IR, waist and hip circumference, waist-to-hip ratio, BMI, body composition, and the percentage reduction in antidiabetic medication use. Safety outcomes were assessed by: (i) adverse events, defined as any undesirable effects attributable to dietary interventions and systematically recorded during the intervention period. These included self-reported symptoms such as headache, dizziness, fatigue, and gastrointestinal disturbances (e.g., bloating or constipation); and (ii) patient-reported hypoglycemic episodes. Reductions in medication use were quantified as a percentage decrease in the dose of diabetes-related medications. Metformin dose reduction was considered when patients exhibited improvement in glycemic control, reflected by fasting blood glucose levels below 130 mg/dL and HbA1c values under 7% at follow-up, accompanied by a reduction in body weight. Upon meeting these criteria, the treating physician exercised clinical judgment to determine the extent of dose reduction, typically between 25–50%, based on the patient’s overall glycemic status and clinical condition [18] Body weight was measured at baseline and every 2 weeks, with the percentage change in weight calculated. HbA1c levels were analyzed at baseline and after 12 weeks. Fasting blood glucose (FBS) was assessed after an overnight fast; while fasting insulin levels were measured to assess insulin sensitivity. Additionally, HOMA-IR was calculated to estimate insulin resistance using fasting glucose and insulin values. BMI, waist, and hip circumferences were recorded at baseline and every 2 weeks. Body composition was evaluated every 2 weeks using a Body Composition Analyzer that employs bioelectrical impedance analysis (BIA) to measure the body’s resistance to a low-level electrical current, allowing for the calculation of fat mass, lean mass, and total body water [19]. Safety was monitored through patient-reported side effects, particularly symptoms of hypoglycemia and headache, alongside regular evaluations, including blood glucose levels, to ensure patient safety.

Adherence to both the fasting protocol and dietary restrictions was closely monitored by the research team at the follow-up visits every 2 weeks. These visits included regular check-ins where participants discussed their progress and challenges. The research team provided feedback, motivational support, and made the necessary adjustments. During these visits, medication adjustments and any adverse events were recorded, and biometric measurements were taken to objectively assess compliance and physiological effects. Additionally, WhatsApp groups and phone calls were used to track adherence between visits.

At baseline, all participants underwent a comprehensive assessment using the Global Physical Activity Questionnaire (GPAQ) to evaluate their baseline physical activity levels [20, 21]. The Global Physical Activity Questionnaire (GPAQ), utilized to assess physical activity levels, evaluates occupational, transport-related, and recreational activities, along with sedentary behavior [20, 21]. It records moderate- and vigorous-intensity activities lasting ≥10 minutes, expressed as Metabolic Equivalent Task (MET) minutes per week (MET-min/week). Activities are standardized at 4 METs for moderate and 8 METs for vigorous intensity. Participants achieving ≥600 MET-min/week are classified as physically active, and those reaching ≥3000 MET-min/week as highly active. Those below 600 MET-min/week are deemed inactive, emphasizing the need for interventions to promote physical activity and reduce health risks [21].

### Sample size calculation

A priori sample size calculation was conducted using G*Power version 3.1.9.2, with the absolute mean difference in HbA1c selected as the primary outcome measure. The effect size (d = 0.51) was calculated based on the absolute mean change in HbA1c reported from a trial by Carter et al. (Diabetes Res Clin Pract, 2019). Based on this effect size, significance level (α = 0.05), and 80% power (β = 0.20), a minimum of 98 participants was required to detect a statistically significant difference in HbA1c between the two groups. Accounting for an anticipated dropout rate of 10%, the final required sample size was increased to 108 participants to ensure adequate statistical power [22, 23].

### Data analysis

All statistical analyses were performed using version 26 of the Statistical Package for the Social Sciences (SPSS) software (IBM Corp., Armonk, NY, USA). Normality testing for the data was conducted using the Shapiro-Wilk and Kolmogorov-Smirnov tests. Descriptive statistics were computed, with continuous variables presented as mean ± standard deviation (SD) for normally distributed data, and as median and interquartile range (IQR) for non-normally distributed variables. Categorical data were summarized as frequency (count) and relative frequency (percentage). Baseline and 12-week characteristics were reported as mean ± SD for continuous variables and as frequencies (percentages) for nominal variables. The final analysis followed a modified intention-to-treat (ITT) approach, including patients who attended at least the first follow-up visit. Missing data were handled using multiple imputation (MI), based on the average of five iterations.

Group comparisons for normally distributed quantitative variables were performed using an unpaired t-test, while the non-parametric Mann-Whitney test was applied to non-normally distributed data. Within-group comparisons of serial measurements were evaluated using paired t-tests or repeated measures analysis of variance (ANOVA) for normally distributed data, and the non-parametric Wilcoxon signed-rank test for non-normally distributed data. Categorical data were compared using the Chi-square (χ²) test, with the Fisher exact test used when expected frequencies were less than 5. A p-value of less than 0.05 was considered statistically significant.

## Results

**Fig. 1:**
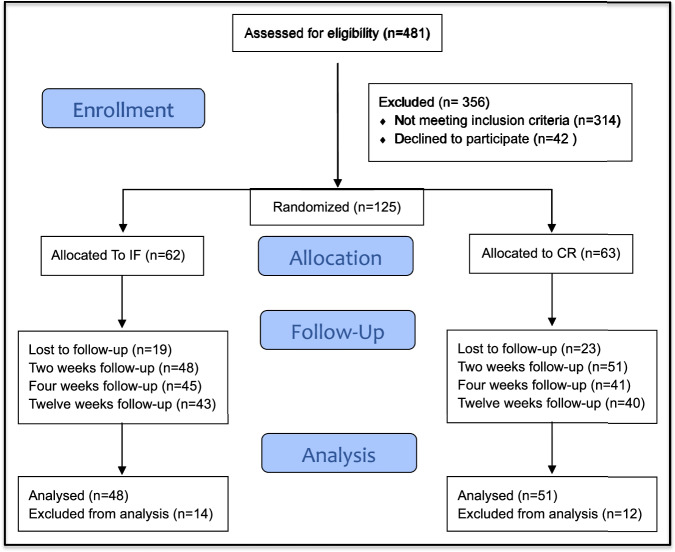
CONSORT flow chart of the study participants. This figure illustrates the flow of participants through each stage of the study. A total of 481 individuals were assessed for eligibility. One hundred twenty-five participants were randomized to either the intermittent fasting (IF) group (*n* = 62) or the calorie restriction (CR) group (*n* = 63). Final analyses included 48 participants in the IF group and 51 in the CR group.

Between November 2021 and April 2024, 481 patients were screened for eligibility, with 99 patients ultimately included in the final analysis (48 in IF + CR and 51 in CR) as shown in Fig. 1. The analysis was based on modified intention-to-treat. Both groups exhibited comparable demographic and clinical characteristics, with hypertension and arthralgia/arthritis being the most prevalent comorbidities. (Fig. 1). There were no significant differences in family history of diabetes or obesity, BMI, waist circumference, or hip circumference between both groups. Physical activity levels were comparable, with average MET minutes of 100 and sedentary hours of 15.7 ( ± 3) hours for IF + CR and 16.2 ( ± 2.3) hours for CR, showing no significant differences (P = 0.134) (Table 1).

**Table 1:** Patient comorbidities and baseline characteristic.

Parameter	IF + CR (N = 48)	CR (N = 51)	*P*-value
**Age (years)**	41.42 ± 9.21	42.00 ± 9.77	0.761^a^
**Height (cm)**	159.10 ± 7.38	158.39 ± 5.88	0.595^a^
**Weight (kg)**	101.70 ± 14.15	104.18 ± 14.04	0.384^a^
**Gender (Female)**	47 (97.9%)	48 (94.1%)	0.618^b^
**Educational level**			0.394^b^
**- University degree**	44 (91.7%)	44 (86.3%)	
**- High school degree**	4 (8.3%)	7 (13.7%)	
**Hypertension**	16 (33.3%)	21 (41.2%)	0.420^b^
**Arthralgia/arthritis**	42 (87.5%)	44 (86.3%)	0.857^b^
**Chronic Heart disease**	2 (4.2%)	4 (7.8%)	0.679^b^
**Chronic Kidney Disease**	1 (2.1%)	2 (3.9%)	>0.999^b^
**Family History of Diabetes**	33 (68.8%)	40 (78.4%)	0.274^b^
**Family History of Obesity**	38 (79.2%)	40 (78.4%)	0.929^b^
**BMI (kg/m²)**	40.22 ± 5.31	41.61 ± 6.00	0.226^a^
**Waist circumference (cm)**	108.77 ± 10.53	111.20 ± 10.04	0.244^a^
**Hip circumference (cm)**	127.40 ± 9.22	128.27 ± 11.07	0.670^a^
**WHR**	0.85 ± 0.07	0.87 ± 0.07	0.298^a^
**RBG (mg/dL)**	154.12 ± 30.89	159.47 ± 35.60	0.428^a^
**HbA1c (%)**	6.96 ± 0.73	7.19 ± 0.95	0.182^a^
**FBG (mg/dL)**	114.17 ± 26.62	114.94 ± 18.80	0.867^a^
**Body fat (%)**	44.80 ± 3.59	44.91 ± 3.67	0.872^a^
**Body water (%)**	38.98 ± 2.37	38.44 ± 2.20	0.244^a^
**Muscle mass (%)**	51.06 ± 3.35	50.67 ± 3.12	0.541^a^
**Bone mass (%)**	3.16 ± 0.19	3.15 ± 0.21	0.744^a^
**Visceral fat (level)**	11.58 ± 2.47	11.90 ± 2.58	0.532^a^
**Lean body mass (kg)**	55.24 ± 4.71	55.92 ± 4.49	0.469^a^
**Body fat mass (kg)**	46.40 ± 9.69	47.35 ± 9.58	0.622^a^
**Bone mass (kg)**	3.15 ± 0.26	3.19 ± 0.24	0.475^a^
**Muscle mass (kg)**	51.16 ± 4.48	52.17 ± 4.10	0.243^a^
**Caloric intake (kcals)**	1493 ± 204	1505 ± 218	0.327^a^
**Antidiabetics used:**			0.363^b^
**- 1 gram of metformin**	12 (25.0%)	12 (25.0%)	
**- 2 grams of metformin**	36 (75.0%)	39 (76.0%)	
**Duration of diabetes**	1.00 [1.00, 2.00]	1.50 [1.00, 5.00]	0.011^c^
**Insulin conc (µIU/mL)**	8.80 [5.85, 12.60]	10.80 [6.70, 17.20]	0.261^c^
**HOMA IR**	2.40 [1.35, 3.30]	3.00 [1.60, 4.90]	0.181^c^
**MET (MIN)**	100 ± 28	100 ± 20	0.495^a^
**Sedentary hours (hours/day)**	15.7 ± 3	16.2 ± 2.3	0.134^a^

*BMI* Body Mass Index, *FBG* Fasting Blood Glucose, *HbA1c* Glycated Hemoglobin, *HOMA-IR* Homeostasis Model Assessment of Insulin Resistance, *IQR* Inter-Quartile Range, *kcals* kilocalories, *MET* Metabolic Equivalent Task, *RBG* Random Blood Glucose, *SD* Standard Deviation, *WHR* Waist to Hip Ratio.

Data are represented as Mean ± SD, Count (%), and Median (IQR).

Level of significance P < 0.05.

^a^Unpaired t-test for between-group comparison.

^b^Chi-square test for categorical data.

^c^Mann-Whitney U test for between-group comparison.

At baseline, the IF + CR group consumed 1493 ± 204 kcals, and the CR group consumed 1505 ± 218 kcals, with no statistically significant difference (p = 0.327). After 12 weeks, the IF group maintained an average caloric intake of 1482 ± 204 kcal, while the CR group slightly reduced intake to 1435 ± 203 kcal; this difference was also not significant (p = 0.669). The use of a personalized nutrition plan ensured that each participant followed an energy deficit tailored to their specific metabolic needs (Tables 1 and 2).

**Table 2:** Study outcomes in both groups after 12-weeks.

Parameter	IF + CR	CR	Between Groups *P*-value
**HbA1c (%)**	6.51 ± 0.67	6.86 ± 0.94	0.035^a^
**FBG (mg/dL)**	104.16 ± 21.18	110.77 ± 18.09	0.098^a^
**Weight (kg)**	94.16 ± 14.32	98.55 ± 13.42	0.118^d^
**BMI (kg/m²)**	37.70 ± 5.53	39.78 ± 5.85	0.072^a^
**Waist circumference (cm)**	105.18 ± 12.18	106.40 ± 12.31	0.621^d^
**Hip circumference (cm)**	123.08 ± 9.01	125.67 ± 10.77	0.200^d^
**WHR**	0.83 ± 0.07	0.83 ± 0.12	0.917^a^
**Body fat (%)**	44.56 ± 8.16	44.24 ± 3.71	0.800^d^
**Body water (%)**	39.45 ± 2.51	38.55 ± 2.13	0.056^d^
**Muscle mass (%)**	51.96 ± 3.03	51.53 ± 3.28	0.502^d^
**Bone mass (%)**	3.21 ± 0.32	3.17 ± 0.23	0.499^d^
**Visceral fat (level)**	10.57 ± 2.09	11.16 ± 2.28	0.184^d^
**Lean body mass (kg)**	52.89 ± 5.21	52.03 ± 4.41	0.060^d^
**Body fat mass (kg)**	42.30 ± 9.48	44.68 ± 9.36	0.212^d^
**Bone mass (kg)**	3.15 ± 0.27	3.16 ± 0.30	0.882^d^
**Muscle mass (kg)**	49.07 ± 5.13	49.15 ± 10.69	0.078^d^
**Caloric intake (kcals)**	1482 ± 204.42	1435 ± 203	0.669^a^
**Insulin Conc (µIU/mL)**	7.25 [4.05, 11.20]	8.40 [6.50, 15.00]	0.263^c^
**HOMA IR**	1.95 [1.15, 2.85]	2.30 [1.40, 4.00]	0.106^c^
**Dose change of Metformin:**	N = 8 (16%)	N = 7 (13%)	0.308^b^
**25% dose reduction**	N = 7	N = 6	
**50% dose reduction**	N = 1	N = 1	

Data are represented as Mean ± SD and Median (IQR).

*BMI* Body Mass Index, *FBG* Fasting Blood Glucose, *HbA1c* Glycated Hemoglobin, *HOMA-IR* Homeostasis Model Assessment of Insulin Resistance, *IQR* Inter-Quartile Range, *kcals* kilocalories, *RBG* Random Blood Glucose, *SD* Standard Deviation, *WHR* Waist to Hip Ratio

^a^Unpaired t-test for between-group comparison.

^b^Chi-Square Test for the categorical data.

^c^Mann-Whitney U test for between-group comparison.

^d^Repeated measure ANOVA for the repeated continuous measurements.

During the 12-week intervention, both groups experienced significant weight reductions. The within-group analysis indicated significant improvements in several parameters for both the IF + CR and CR groups, including weight, BMI, hip circumference, HbA1c, body fat mass, visceral fat, muscle mass, lean body mass, and HOMA-IR. Notably, the IF + CR group exhibited additional significant changes in fasting blood glucose and insulin concentration, whereas the CR group achieved significant reductions in waist circumference and body fat percentage (Tables 2 and 3).

**Table 3:** Percentage change of the patients’ characteristics in the two treatment groups represented as median [IQR].

Parameter	IF + CR(N = 48)	CR(N = 51)	*P*-value
**HBA1C % change**	−6.94 [−8.84 to −4.79]	−3.08 [−6.06−to −1.47]	0.001^a^
**FBG % change**	−5.79 [−10.44 to −1.02]	−3.20 [−10.43 to 4.00]	0.161^a^
**Weight % change**	−6.51 [−9.56 to −5.21]	−4.41 [−5.98 to −3.64]	<0.001^a^
**Waist circumference % change**	−4.64 [−7.44 to −2.62]	−2.70 [−5.31 to −0.79]	0.020^a^
**Hip circumference % change**	−3.12 [−4.63 to −1.18]	−0.86 [−3.10 to 0.00]	0.013^a^
**WHR % change**	−2.60 [−4.74 to −0.43]	−1.67 [−4.20 to 0.00]	0.281^a^
**Body fat % change**	−3.18 [−4.49 to −0.69]	−1.32 [−3.61 to −0.22]	0.060^a^
**Body water % change**	0.66 [0.00 to 2.09]	0.25 [−1.27 to 1.58]	0.060^a^
**Muscle mass % change**	2.76 [0.95 to 3.73]	1.60 [−0.23 to 3.31]	0.188^a^
**Bone mass % change**	0.00 [0.00 to 3.28]	0.00 [0.00 to 3.12]	0.424^a^
**Visceral fat level % change**	−9.09 [−14.29 to 0.00]	−7.14 [−11.11 to 0.00]	0.094^a^
**Lean body mass % change**	−3.49 [−5.94 to −1.76]	−3.10 [−3.66 to 0.00]	0.079^a^
**Body fat mass % change**	−9.39 [−13.74 to −6.27]	−5.32 [−8.91 to −1.61]	<0.001^a^
**BMI % change**	−5.93 [−7.94 to −4.78]	−4.08 [−5.54 to −2.63]	<0.001^a^
**Insulin concentration % change**	−7.38 [−25.32 to 1.58]	−6.11 [−33.33 to 4.44]	0.820^a^
**HOMA IR % change**	−11.65 [−31.37 to 0.00]	−9.48 [−23.33 to 0.00]	0.549^a^

Data are represented as Median [IQR].

Significance level: P < 0.05.

*BMI* Body Mass Index, *FBG* Fasting Blood Glucose, *HOMA-IR* Homeostasis Model Assessment of Insulin Resistance, *IQR* Inter-Quartile Range, *SD* Standard Deviation, *WHR* Waist to Hip Ratio.

^a^Mann-Whitney U test used for between-group comparison.

**Fig. 2:**
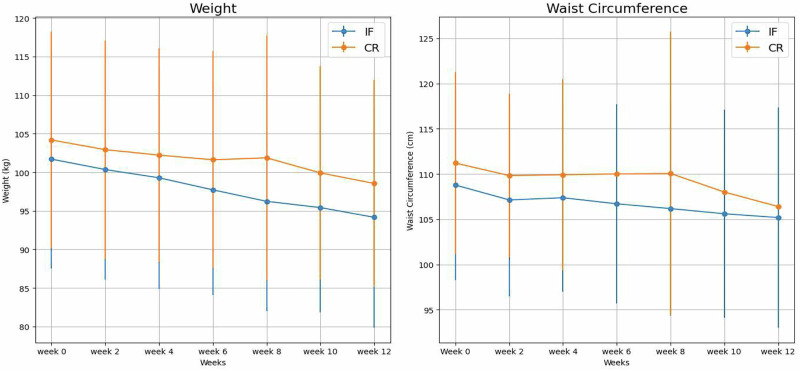
Changes in weight and waist circumference over 12 weeks. Left Panel: Weight (kg). Mean ± SD weight values across all follow-up weeks. At week 12, weight was 94.16 ± 14.32 kg in the IF+CRgroup and 98.55 ± 13.42 kg in the CR group (*p* = 0.118). Right Panel: Waist Circumference (cm). Mean ± SD waist circumference values for IF+CR and CR groups throughout the 12-week intervention. At week 12, waist circumference was 105.18 ± 12.18 cm in the IF+CR group and 106.40 ± 12.31 cm in the CR group (*p* = 0.621). Between group comparisons over time were analyzed using repeated measures ANOVA.

Comparing both groups at the end of the study, there were no significant differences in the parameters measured except for HbA1c. However, comparing the percentage change in parameters from baseline showed that there are significant differences between both groups. The IF + CR group demonstrated a significant reduction in HbA1c, achieving a mean level of 6.51% compared with 6.86% in the CR group, with P = 0.035. Analysis of the absolute reduction in HbA1c showed a significantly greater median decrease in the IF + CR group [−0.50 (IQR − 0.60 to −0.35)] compared with the CR group [−0.20 (IQR − 0.40 to −0.10)] (P = 0.002), confirming the higher glycemic improvement with the IF + CR intervention. In terms of percentage change of HbA1c from baseline, the IF + CR group exhibited a greater median percentage reduction of –6.94% (IQR –8.84 to –4.79) versus –3.08% (IQR –6.06 to –1.47) in the CR group), with P = 0.001. The IF + CR group showed a more pronounced weight reduction (-6.51%) compared to the CR group (-4.41%), with a P < 0.001.Moreover, the IF + CR group demonstrated a significantly greater percentage change in waist circumference (-4.64%) and hip circumference (-3.12%) compared to the CR group (-2.70% and -0.86%, respectively), with P-values of 0.020 and 0. 013 (Fig. 2) (Tables 2 and 3).

The body fat percentages of both the IF + CR and CR groups were significantly higher than the healthy range (21–33% for females and 8–24% for males), representing obesity and an increased risk of metabolic and cardiovascular diseases [24]. Body composition changes revealed that the IF + CR group had a significant reduction in body fat mass (-9.39%) compared to the CR group (-5.32%), with a P < 0.001. Both groups experienced significant reductions in BMI, with the IF + CR group achieving a greater decrease (-5.93%) compared to the CR group (-4.08%). The difference between the groups was statistically significant (p-value < 0.001). Both groups also showed improvements in fasting blood glucose (FBG), HbA1c, insulin levels, and HOMA-IR. Overall, both interventions significantly changed weight, body composition, and metabolic parameters, with the IF + CR group showing slightly more pronounced changes in certain areas (Table 3).

Both dietary regimens, IF and CR, were well-tolerated overall, with no severe side effects reported. Mild headaches were reported by seven participants overall, with four in the IF + CR group and three in the CR-only group but resolved spontaneously and did not necessitate intervention or discontinuation of the dietary intervention. Notably, no cases of hypoglycemia were reported in either group, demonstrating the safety of both dietary interventions in managing blood glucose levels.

## Discussion

This 3-month randomized clinical trial revealed that a 12-hour night intermittent fasting (IF) regimen combined with a calorie-restricted diet (IF-CR) is more effective in promoting weight loss and reducing body fat mass than a calorie-restricted diet alone in people living with type 2 diabetes mellitus. Additionally, the IF + CR group showed a significant improvement in HbA1c levels compared to the CR group, indicating a more pronounced effect on glycemic control. However, both dietary interventions were equally effective in improving insulin resistance.

In our study, the absolute median reduction in HbA1c was also significantly greater in the IF + CR group ( − 0.50 [IQR − 0.60 to −0.35]) compared with the CR group ( − 0.20 [IQR − 0.40 to −0.10]; P = 0.002), indicating greater short-term glycemic improvement with the combined intervention. While the reduction was statistically significant, its clinical impact is likely to become more apparent with extended intervention durations, considering that HbA1c represents mean glucose control over roughly a three-month period.

Our study observed a 16% (N = 8) reduction in medication use in the (IF + CR) group, compared to a 13% (N = 7) reduction in the CR group, but the difference between groups was not statistically significant (P = 0.308). Che et al. noted medication dose reductions in a 10-hour IF protocol [25]. Andriessen et al. did not assess or report the effects on medication use [26] Sepúlveda B et al. found that both early and late (IF) protocols contributed to positive glycemic control. However, the study did not provide data on medication use or reductions in medication doses [27].These studies indicate that (IF) has a positive effect on glycemic control, without focusing on the magnitude or percentage reductions in medication use [25–29, 30]. The statistically non-significant difference between IF + CR and CR groups in our study suggests comparable magnitudes of reduction, a point not thoroughly explored in previous studies [26, 31].

Our findings align with several other clinical trials that have investigated the effects of night IF on weight loss and body composition in T2D patients [25, 28, 29, 32]. For instance, Che et al. demonstrated that 12 weeks of 10-hour IF resulted in a 3% reduction in body weight among people living with obesity and T2D [25]. Similarly, Andriessen et al. reported a 1.1% weight loss after 3 weeks of 14-hour IF in a small cohort of men and women with obesity and T2D [26]. The weight loss observed in our study (6.51% from baseline) was greater than these previous reports, likely due to both calorie restriction and the intervention period of 12 weeks.

The significant reduction in HbA1c levels in the IF + CR group (6.51 ± 0.67%) compared to the CR group (6.86 ± 0.94%) with a P = 0.035 is consistent with findings from other studies [28, 29, 32]. Few studies found that calorie restriction, with or without IF, improved HbA1c levels in overweight people living with type 2 diabetes mellitus [33].

Both the IF + CR and CR groups showed improvements in insulin resistance (HOMA-IR), although the difference was not statistically significant. This is in line with the findings of Andriessen et al., who reported that 3 weeks of time-restricted eating improved glucose homeostasis but did not significantly improve insulin sensitivity [26]. The lack of significant difference in insulin resistance between the IF + CR and CR groups in our study may be due to the relatively short duration of the intervention. This is consistent with the findings of other studies [26, 28].

The observed discrepancies between our study and other clinical trials can be attributed to several critical factors. Firstly, the 12-week intervention period in our study, which exceeds the duration of some other studies, contributed to more substantial weight loss and significant improvements in HbA1c levels. Secondly, the specific 12-hour fasting window employed in our study may have been more efficacious in enhancing weight loss and glycemic control compared to the shorter fasting windows utilized in other studies [33]. Lastly, the provision of individualized diabetes nutrition counseling to both groups in our study may have played a pivotal role in the observed improvements in glycemic control and adherence.

Our study’s inclusion criteria and caloric estimation methodology were carefully designed to align with recent studies on IF and CR [25, 27, 33]. Unlike many studies that focus solely on IF or CR, our approach combined CR with a 12-hour daily fasting period. This structured regimen may contribute to more consistent metabolic improvements, as observed in our cohort. Our study used the Global Physical Activity Questionnaire (GPAQ) to systematically assess physical activity levels [21]. Comparable studies often lack such rigorous physical activity tracking, which we believe is crucial for understanding the full impact of nutrition interventions on metabolic health [11, 25, 26, 29].

When examining participant characteristics, our study’s age, gender, and BMI distributions were comparable to those reported in other studies [10, 11]. This similarity enhances the generalizability of our findings to middle-aged adults with obesity and T2D. However, our predominantly female sample and short diabetes duration may account for some differences in outcomes. The relatively short duration of diabetes among our study participants may have contributed to the observed improvements in glycemic control, as newly diagnosed cases are generally more responsive to interventions compared to long-standing cases with established insulin resistance.

In terms of comorbidities, the prevalence of hypertension in our study was consistent with other IF studies, reinforcing the relevance of our findings [31]. IF may enhance metabolic parameters, improve insulin sensitivity, and induce favorable hormonal changes, such as increased adiponectin and reduced leptin levels, which collectively contribute to better weight management and glycemic control [25, 28, 29, 33]. These physiological adaptations could explain the greater improvements observed in the IF + CR group.

Clinically, our findings suggest that incorporating (IF) into current dietary guidelines for managing Type 2 Diabetes (T2D) could offer significant benefits over calorie restriction (CR) alone. Our study demonstrated that the combination of IF and CR resulted in greater improvements in glycemic control, weight management, and overall health outcomes compared to CR alone. These findings highlight the potential of IF as an effective adjunct to traditional dietary approaches, making it an attractive option for both patients and healthcare providers.

Patient-centered outcomes, including qualitative data on the acceptability and sustainability of (IF), are critical for tailoring effective dietary interventions [34]. Understanding patient preferences and lifestyle factors can play a significant role in the success of such strategies [35]. While our study suggests potential benefits of IF based on clinical outcomes, measures such as quality of life or acceptance of IF were not assessed, highlighting an area for further research to better understand patient experiences and preferences [34, 35].

### Limitations

Blinding participants was not feasible as they needed to fully understand the intervention, which could introduce bias in self-reported outcomes and adherence. Although physical activity was monitored throughout the study, it was not assessed at the end. Another limitation of our study is the predominance of female participants, which likely reflects the greater tendency of women to participate in dietary and lifestyle interventions in real-world settings and may restrict the generalizability of the findings to male populations. In addition, the relatively short duration of diabetes among participants could have enhanced their responsiveness to lifestyle interventions. Furthermore, the study did not evaluate psychological factors such as stress, mood, and eating behaviors, which can influence adherence and outcomes. Also, leptin, adiponectin, and other metabolic markers were not measured, which limits the study’s ability to understand the underlying biological mechanisms driving the effect of the interventions. Including these assessments could provide a more comprehensive understanding of the interventions’ impact.

## Conclusion

This study demonstrates that a 12-hour IF regimen combined with a calorie-restricted diet is more effective for weight loss and reducing body fat mass compared to a calorie-restricted diet alone in people living with T2D. Additionally, the IF + CR group showed a significant improvement in HbA1c levels compared to the CR group, indicating a more pronounced effect on glycemic control. Integrating IF into dietary guidelines could enhance patient outcomes and adherence, offering a promising approach to diabetes management. Further research is needed to explore the long-term effects and adherence to IF in this population.

## Data Availability

All the study data will be available on reasonable request to the corresponding author.
